# Beyond Dopamine: A Review of KarXT (Xanomeline-Trospium) as a Novel M1/M4 Muscarinic Agonist for Schizophrenia

**DOI:** 10.1192/j.eurpsy.2026.12187

**Published:** 2026-07-31

**Authors:** C. Cañete, C. Andres, C. Lopez, I. Salmerón, G. Cabeza

**Affiliations:** Psychiatry, Hospital Clinico de Barcelona, Barcelona, Spain

## Abstract

**Introduction:**

The therapeutic landscape of schizophrenia has been dominated by D2 receptor antagonists for decades. While effective for psychosis, this mechanism incompletely addresses cognitive and negative symptoms, which are primary drivers of poor functional outcomes. Furthermore, the significant metabolic and neurological side-effect burden of current agents leads to high rates of non-adherence. KarXT (xanomeline-trospium), an M1/M4 muscarinic agonist, represents the first non-dopaminergic mechanism to demonstrate robust efficacy for psychosis in pivotal trials.

**Objectives:**

The objective of this review is to critically evaluate the evidence for KarXT, focusing on its mechanism, clinical efficacy from recent Phase 3 trials, and its distinct safety profile. We specifically assess its potential as an alternative strategy to D2-based therapies and its implications for addressing the unmet needs of patients with schizophrenia.

**Methods:**

A targeted narrative review was conducted using PubMed and ClinicalTrials.gov (2020-2025) for ’KarXT’, ’xanomeline’, ’M1/M4’, and ’schizophrenia’. The review prioritized data from the pivotal EMERGENT program (Phase 3 RCTs) and key Phase 2 studies that defined the therapeutic window and safety parameters.

**Results:**

KarXT’s efficacy is driven by M1/M4 agonism in the CNS, bypassing D2 blockade. To mitigate peripheral cholinergic effects, it is co-formulated with the peripherally-restricted antagonist trospium. The EMERGENT trials met their primary endpoints, demonstrating significant PANSS total score reductions versus placebo. Crucially, KarXT was not associated with weight gain, metabolic dysfunction, or extrapyramidal symptoms (EPS). Gastrointestinal adverse events (nausea, vomiting) were the most common, primarily mild-to-moderate and transient, typically decreasing after the initial titration phase.

**Conclusions:**

KarXT represents the first major therapeutic advance in schizophrenia not based on D2 antagonism, offering a fundamentally new treatment modality. Its unique profile—robust efficacy without the metabolic or neurologic side effects of current standards—positions it as a critical new option for a broad range of patients, especially those intolerant to existing agents or with residual symptoms. This development confirms the muscarinic system as a viable, non-dopaminergic target for psychosis.

**Disclosure of Interest:**

None Declared

